# The role and therapeutic potential of heat shock proteins in haemorrhagic stroke

**DOI:** 10.1111/jcmm.14479

**Published:** 2019-07-05

**Authors:** Anwen Shao, Yunxiang Zhou, Yihan Yao, Wenhua Zhang, Jianmin Zhang, Yongchuan Deng

**Affiliations:** ^1^ Department of Neurosurgery, The Second Affiliated Hospital, School of Medicine Zhejiang University Hangzhou China; ^2^ Department of Surgical Oncology, The Second Affiliated Hospital, School of Medicine Zhejiang University Hangzhou China; ^3^ Department of Neurology The Second Affiliated Hospital School of Medicine Zhejiang University Hangzhou China

**Keywords:** heat shock proteins, intracerebral haemorrhage, review, stroke, subarachnoid haemorrhage, therapeutic target

## Abstract

Heat shock proteins (HSPs) are induced after haemorrhagic stroke, which includes subarachnoid haemorrhage (SAH) and intracerebral haemorrhage (ICH). Most of these proteins function as neuroprotective molecules to protect cerebral neurons from haemorrhagic stroke and as markers to indicate cellular stress or damage. The most widely studied HSPs in SAH are HSP70, haeme oxygenase‐1 (HO‐1), HSP20 and HSP27. The subsequent pathophysiological changes following SAH can be divided into two stages: early brain injury and delayed cerebral ischaemia, both of which determine the outcome for patients. Because the mechanisms of HSPs in SAH are being revealed and experimental models in animals are continually maturing, new agents targeting HSPs with limited side effects have been suggested to provide therapeutic potential. For instance, some pharmaceutical agents can block neuronal apoptosis signals or dilate cerebral vessels by modulating HSPs. HO‐1 and HSP70 are also critical topics for ICH research, which can be attributed to their involvement in pathophysiological mechanisms and therapeutic potential. However, the process of HO‐1 metabolism can be toxic owing to iron overload and the activation of succedent pathways, for example, the Fenton reaction and oxidative damage; the overall effect of HO‐1 in SAH and ICH tends to be protective and harmful, respectively, given the different pathophysiological changes in these two types of haemorrhagic stroke. In the present study, we focus on the current understanding of the role and therapeutic potential of HSPs involved in haemorrhagic stroke. Therefore, HSPs may be potential therapeutic targets, and new agents targeting HSPs are warranted.

## 1 INTRODUCTION

Stroke, which is the second leading cause of fatality worldwide and the primary cause of permanent disability among adults, includes ischaemic stroke and haemorrhagic stroke.^1^, ^2^ The latter accounts for approximately 10%‐20% of all strokes yet, have a higher mortality rate vs the former.^3^, ^4^, ^5^, ^6^ Furthermore, subarachnoid haemorrhage (SAH) and intracerebral haemorrhage (ICH) constitute haemorrhagic strokes, with worldwide incidences of 9.1 and 24.6 per 100,000 person years respectively.^4^, ^7^, ^8^ Despite increasing attention on the prevention of stroke and new methods for treating intracranial haemorrhage, incidences of SAH and ICH and the case fatality rate of ICH have not decreased over time.^7^, ^8^, ^9^ Additionally, even if patients survive, more than half of haemorrhagic stroke survivors never recover enough brain function to live on their own, which is attributed to the pathophysiological processes following haemorrhage.^1^, ^10^, ^11^ Thus, it is necessary to explore the pathophysiological mechanisms of haemorrhagic stroke to seek potential treatments.

**Figure 1:**
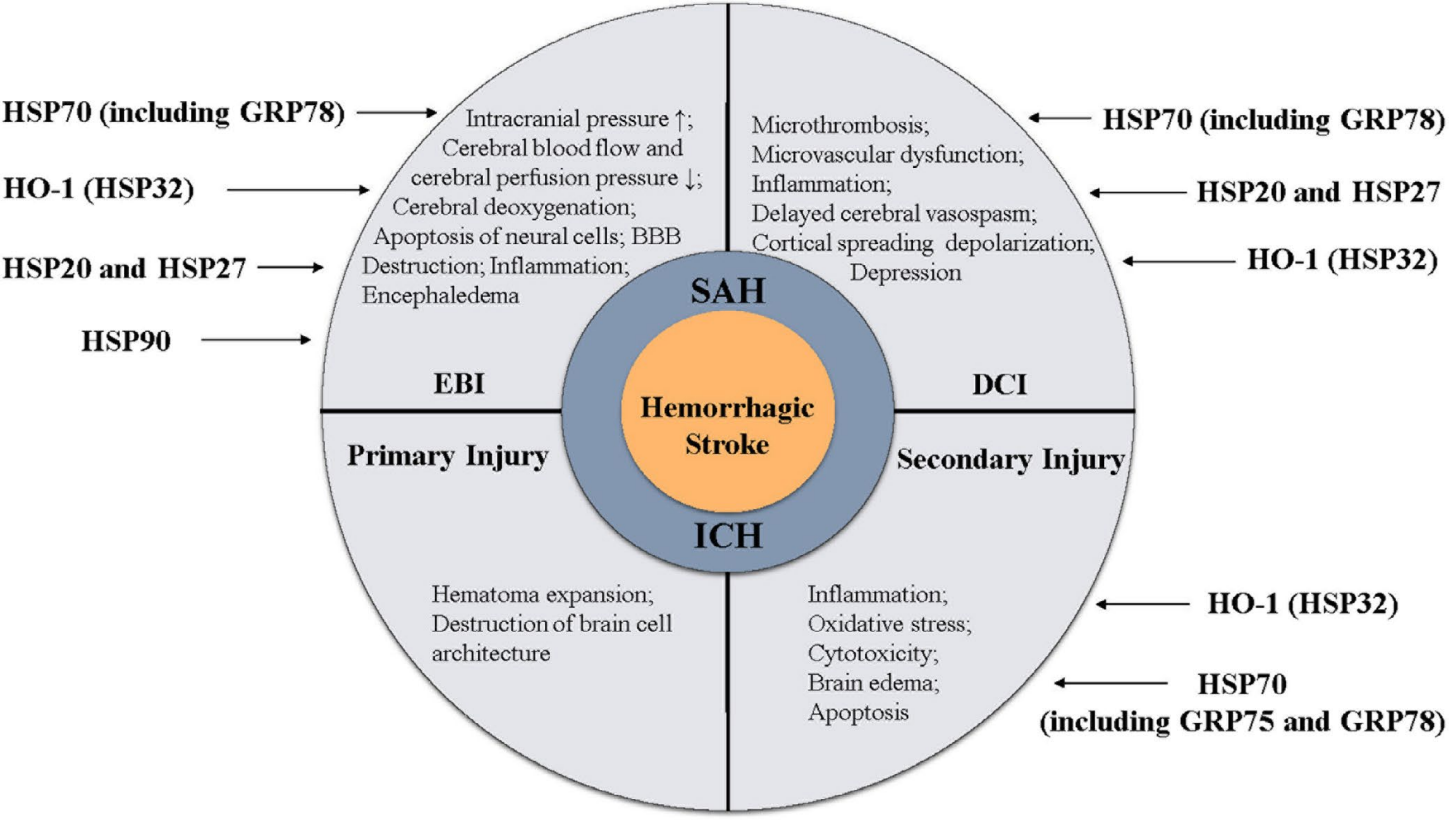
Schematic representation of the relationship between heat shock proteins and haemorrhagic stroke

Heat shock proteins (HSPs), a family of evolutionarily conserved molecular chaperones that includes HSP90, HSP70, HSP60, HSP40 and small heat shock proteins (sHSPs), which have various functions in proteostasis, are defined by the presence of heat shock elements in their promoters.^12^, ^13^ These proteins participate in pathophysiological neurological mechanisms by regulating stress responses, mitigating apoptotic signals, stabilizing the cytoskeleton and shuttling damaged proteins for degradation via the ubiquitin‐proteasome system or autophagy (Table 1).^12^, ^14^, ^15^ Therefore, the functional roles of HSPs may be potential targets for haemorrhagic stroke therapeutics (Figure 1). To explore the explicit mechanisms of HSPs in haemorrhagic stroke, several experimental models in animals have been established over time.

**Table 1:** HSPs involved in haemorrhagic stroke

**HSPs**		**Pathophysiological processes**	**Potential mechanisms**	**The induction location**	**Effect and roles**	**Related agents**	**Reference**
*Subarachnoid haemorrhage*							
HSP70 (including GRP78)		Cerebral vasospasm, neural cell apoptosis, immunoreaction and inflammation	Refold protein, degrade damaged proteins, inhibit apoptosis, mediate BBB disruption and cell death via aberrant proteolysis	Bilateral neocortex, hippocampus, thalamus, septum, hypothalamus, caudoputamen and basal forebrain	Neuroprotective molecule, significant marker for cellular stress or damage; crucial predictor of poor prognosis; blood biomarker for the early differential diagnosis of haemorrhagic stroke and ischemic stroke	Geranylgeranylacetone, modified HSP70 proteins (eg TAT‐Hsp70), valproic acid, atorvastatin,	12, 14, 15, 16, 17, 22, 24, 25, 28, 31, 33, 34, 35, 36, 37, 38, 40, 41, 50, 51, 52, 53, 54, 55, 56, 57, 58, 96, 97, 98
HO‐1		Cerebral vasospasm, lipid peroxidation	Metabolize haeme, remove haeme and iron, diastolic vascular smooth muscle^a^	Microglia and cerebral blood vessels	Possible neuroprotective molecule^b^; marker of cellular stress and damage in infarcted regions	Nicaraven, argon, carnosol, ebselen and CGS26393, HO‐1 protein combined with protein transduction domains	14, 39, 40, 61, 62, 63, 64, 65, 66, 70, 71, 99, 100, 101
HSP20 and HSP27		Cerebral vasoconstriction, apoptosis pathway	Solubilize misfolded proteins and hinder their aggregation, suppress cell death signaling and protect neurons against ischemic injury	Astrocytes in the ischaemic zone and the ischaemic penumbra	Important molecules in cerebral vasoconstriction without ATP necessarily involved in the function	AZX100	14, 28, 40, 73, 74, 75, 76, 77, 78
HSP90		Apoptosis, inflammation, and BBB destruction	Stabilize and promote the function of P2X7 receptor, which is abundant in the nervous system and is associated with the pathophysiological process of inflammation and oxidative stress in EBI	Microglia and neurons of the hippocampus	Neurotoxic factor in the development of EBI	17‐allylamino‐17‐demethoxygeldanamycin, A438079	79, 80, 81, 82, 83, 84, 85
*Intracerebral haemorrhage*							
HO‐1		Inflammation, oxidative stress and cytotoxicity	Increase oxidative stress, accelerate the accumulation of iron overload, promote inflammation and increase secondary injury	Microglia and cerebral blood vessels	Possible harmful molecule^b^	Haemin, nicotinamide mononucleotides, minocycline	14, 20, 115, 116, 117, 118, 119, 120, 121, 122, 123, 124, 125, 126, 134, 144, 145, 146
HSP70	GRP75	Neural cell apoptosis, immunoreaction	Inhibit inflammation and neuronal apoptosis	Mainly located in mitochondria, reduced after intracerebral haemorrhage	Neuroprotective molecule	Minocycline, geranylgeranylacetone, geldanamycin, Di Dang Tang	36, 135, 136, 137, 138, 139, 140, 147, 148, 149, 150
	GRP78	Neural cell apoptosis	Inhibit neuronal apoptosis	Mainly located in endoplasmic reticulum	Neuroprotective molecule		

Abbreviations: BBB, blood‐brain barrier; EBI, early brain injury; GRP75, glucose‐regulated protein 75; GRP78, glucose‐regulated protein 78; HO‐1, haeme oxygenase‐1; HSP, heat shock protein; TAT, N‐terminal transactivator of transcription.

a Carbon monoxide, one of the haeme metabolite, can up‐regulate soluble guanylyl cyclase, which contributes to cyclic guanosine monophosphate accumulation and subsequently leads to vascular smooth muscle relaxation.

b The role of HO‐1 is still controversial, the overall effect of HO‐1 in SAH and ICH tends to be protective and harmful respectively.

Among all the SAH models, endovascular perforation in small animals is optimal, as the subsequent responses including changes in haemodynamics and the sudden elevation of intracranial pressure parallel human aneurysmal SAH better than autologous blood injection.^16^, ^17^ However, some studies have demonstrated that the double haemorrhage model, in which SAH is induced a second time 48 hours after the first autologous blood injection, directly corresponds with the time course of cerebral vasospasm in humans.^18^, ^19^ For ICH, disease models made by collagenase or autologous blood injection are both options, and each has its own pros and cons.^20^ With the gradual improvement of animal models, we have discovered more HSP functions in SAH and ICH. In the present review, we will summarize and integrate research findings on the role of HSPs in haemorrhagic stroke and their therapeutic potential.

## 2 THE PATHOPHYSIOLOGY OF SAH

Subarachnoid haemorrhage constitutes 5%‐10% of all strokes and is mainly (85%‐98.9%) caused by ruptured aneurysms.^21^, ^22^, ^23^, ^24^, ^25^, ^26^ Typically, the basic pathophysiological changes during SAH can be divided into two periods: early brain injury (EBI) and delayed cerebral ischaemia (DCI).^27^ The former takes place within the first 3 days of SAH, while the latter usually appears 3‐4 days after the aneurysm rupture and usually resolves after 12‐14 days.^22^, ^28^ The concept of DCI, which consists of delayed cerebral vasospasm and subsequent microvascular dysfunction, microthrombosis (especially in the cortical vessels), inflammation, cortical spreading depolarization, depression and ischaemia, was proposed over 150 years ago.^22^ Cerebral vasospasm was once considered to be a prerequisite for DCI; however, DCI can develop without angiographic vasospasms, and vasospasms may also end with no ischaemic lesions.^29^, ^30^ The EBI stage was proposed more recently and involves elevated intracranial pressure, decreased cerebral blood flow and cerebral perfusion pressure, cerebral deoxygenation, apoptosis of neural cells, destruction of the blood‐brain barrier (BBB), inflammation and encephaledema.^16^, ^22^, ^24^, ^31^, ^32^ The pathophysiological changes occurring during EBI might induce the development of DCI, but this has not yet been confirmed.^22^, ^33^, ^34^

### 2.1 The role of HSP70 plays in SAH

Present in all cells, constitutively active HSP70 is usually called HSC70 or HSP73, while inducible HSP70 protein, commonly called HSP72 or HSP70, is barely detectable in normal brain tissues but becomes the abundant protein when heat shock, ischaemia or other injuries occur.^14^ As the most widely studied HSP, HSP70 is known to be involved in protein refolding and the degradation of damaged proteins.^24^, ^35^, ^36^ HSP70 is involved in the development of SAH by participating in EBI and DCI, and acts as a critical molecule in cerebral vasospasm, neural cell apoptosis, immunoreaction and inflammation.^14^, ^16^, ^17^, ^28^, ^36^, ^37^, ^38^ HSP70 might be a potential target for SAH treatment and management, especially because no strategies explicitly targeting SAH have been developed yet.^39^ Additionally, HSP70 is a significant marker for cellular stress or damage and is also a crucial predictor of poor prognosis as it indicates the severity and extent of brain injury during SAH.^16^, ^17^, ^31^, ^36^, ^38^, ^40^ Moreover, because it is expressed at lower levels in haemorrhagic stroke than in ischaemic stroke, HSP70 can be used as a blood biomarker for the early differential diagnosis of haemorrhagic stroke and ischaemic stroke.^36^, ^41^

#### 2.1.1 HSP70 and early brain injury

Although many efforts have been made to explore the therapeutic targets of delayed cerebral vasospasm, all of the new drugs found in recent years have failed to improve the clinical outcome of patients with SAH.^22^, ^37^ As discussed previously, the development of DCI is not caused solely by delayed cerebral vasospasm, and there might be causality between EBI and DCI; thus, agents directed at treating DCI would not eliminate the root causes of DCI, and this may explain the frustrating results from new drugs to treat SAH.^22^, ^33^ The alleviation of delayed vasospasm does not improve the clinical outcome in patients with SAH, which leads to a reasonable doubt as to whether delayed cerebral vasospasm affects the prognosis of SAH.^22^, ^24^, ^31^, ^40^, ^42^, ^43^, ^44^ Therefore, an increasing number of scholars have begun to support the theory that EBI is the chief cause of mortality and disability following SAH.^24^, ^45^, ^46^

Brain stress and acute ischaemia produced by elevated intracranial pressure, decreased cerebral blood flow and cerebral perfusion pressure can induce the expression of HSP70 4 hours after haemorrhage.^16^, ^36^ As the most common mechanism in EBI, apoptosis increases the permeability of the BBB and encephaloedema and decreases cerebral blood flow.^31^ It is well known that HSP70 is involved in many apoptotic mechanisms; however, related studies involving SAH are scant.^14^, ^29^ The results from literature concerning EBI after SAH showed that HSP70 could inhibit apoptosis via the regulation of the phosphorylation of anti‐apoptotic Akt kinase, which plays a critical role in EBI.^24^ Moreover, HSP70 can decrease the activity of matrix metalloproteinase‐9, which is a member of the zinc endopeptidase family, and is able to mediate BBB disruption and cell death via aberrant proteolysis.^24^, ^47^, ^48^ HSP70 contributes to the decrease of inflammation and cerebral oedema during EBI.^14^, ^36^

**Figure 2:**
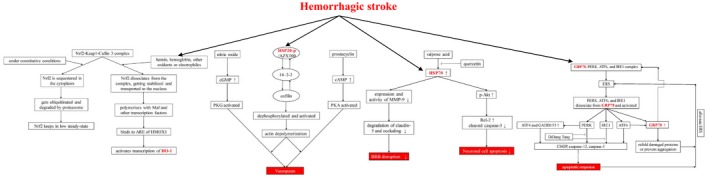
The potential mechanism of heat shock proteins involved in haemorrhagic stroke. PERK, protein kinase RNA‐like ER kinase; AFT6, activating transcription factor 6; IRE1, inositol‐requiring protein 1α; ERS, endoplasmic reticulum stress; AFT4, activating transcription factor 4; GADD153, growth arrest and DNA‐damage‐inducible gene 153; CHOP, C/EBP homologous protein; PKG, cGMP‐dependent protein kinase; PKA, cAMP‐dependent protein kinase; HSP20‐p, HSP20 phosphopeptide; BBB, blood‐brain barrier; MMP‐9, matrix metalloproteinases 9; p‐Akt, phosphorylated RAC‐alpha serine/threonine‐protein kinase; Bcl‐2, B‐cell lymphoma 2; Nrf2, nuclear factor erythroid‐2 related factor 2; Keap1, actin‐bound Kelch like‐ECH‐associated protein 1; ARE, antioxidant response elements; HMOX1, haeme oxygenase (decycling) 1

Endoplasmic reticulum (ER) stress is believed to exert important roles in EBI.^46^, ^49^, ^50^ Glucose‐regulated protein 78 (GRP78), which belongs to HSP70 family, is mainly localized in the ER^51^ Acting as a chaperone that folds and transports proteins, GRP78 can bind to three ER sensors, protein kinase RNA‐like ER kinase (PERK), activating transcription factor 6 (ATF6), and inositol‐requiring protein 1α (IRE1).^31^ The complexes are stable under normal cell conditions while decomposed under ER stress^31^. Unfolded proteins accumulate in the ER when the protein folding or processing reaction is impaired during EBI pathology, triggering the unfolded protein response (UPR) to alleviate ER stress.^31^, ^49^ The UPR signal is transmitted to cytoplasm and nucleus by decomposed GRP78 and subsequently gets initiated; PERK, ATF6 and IRE1 simultaneously dissociate from GRP78 and get activated to modulate UPR.^49^, ^52^, ^53^ Activated ATF6 subsequently increases GRP78 expression and up‐regulated GRP78 can in turn bind to and stabilize free PERK, ATF6 and IRE1 and alleviates ER stress by refolding damaged proteins and preventing them from aggregating.^31^, ^49^, ^54^ However, once UPR fails to control the extent of ER stress, downstream effectors such as C/EBP homologous protein (CHOP) and caspase‐12 can be activated by PERK, ATF6 and IRE1 pathways, and the apoptotic response is triggered (Figure 2).^55^, ^56^

#### 2.1.2 HSP70 and delayed cerebral vasospasm

Although it does not always occur and can be asymptomatic, delayed cerebral vasospasm plays a vital role in the outcome and prognosis of SAH.^29^, ^30^, ^37^, ^57^, ^58^, ^59^ Of patients with delayed cerebral vasospasm, 26%‐38% patients suffer sequelae and die despite undergoing satisfactory surgery to treat aneurysm and perioperative treatment.^37^, ^60^ Cerebral vasospasm was once considered the leading preventable cause of mortality and morbidity in patients with aneurysmal SAH.^29^ However, HSPs are likely to protect neural cells.^14^ Thus, many experimental studies have explored the mechanism and potential therapeutic value of HSPs in the treatment of delayed cerebral vasospasm.^22^, ^37^ HSP70 induction can be seen in the bilateral neocortex, hippocampus, thalamus, septum, hypothalamus,caudoputamen and basal forebrain 5 days after SAH.^16^, ^17^ It is known that HSP70 is highly sensitive to vasospasm, and immunostaining with HSP70 is more sensitive than histological examination for the detection of ischaemia and injury after SAH; it is even possible to build a quantifiable model of nerve damage in rats based on HSP70 sensitivity to vasospasm.^16^, ^17^, ^37^ However, the definite pathways involved in HSP70 induction caused by delayed cerebral vasospasm have remained unclear until now.^17^, ^38^

### 2.2 The roles that Haeme oxygenase‐1 (HO‐1), sHSPs and HSP90 play in SAH

#### 2.2.1 The role HO‐1 plays in SAH

haeme oxygenase‐1, also known as HSP32, can be induced in microglia and cerebral blood vessels by the presence of haeme itself after brain haemorrhage and metabolizes haeme to ferrous iron, biliverdin and carbon monoxide (CO).^14^, ^61^, ^62^, ^63^ After aneurysm rupture, lysed red blood cells can trigger pathophysiological cascades including cerebral vasospasm and lipid peroxidation that can be attenuated by HO‐1 via the enhancement of haeme clearance, which intensifies iron sequestration and increases the levels of the antioxidant bilirubin.^40^, ^64^, ^65^, ^66^In addition, CO can up‐regulate soluble guanylyl cyclase, which contributes to cyclic guanosine monophosphate (cGMP) accumulation and subsequently leads to vascular smooth muscle relaxation.^64^, ^65^ However, bilirubin oxidation products have been implicated in vasospasm and clinical complications following SAH.^67^, ^68^ Thus, HO‐1 may simultaneously play a protective and harmful role in SAH. However, the overall effect of HO‐1 has been shown to be neuroprotective.^14^, ^39^, ^63^, ^64^, ^65^, ^66^, ^69^ Additionally, the overexpression of HO‐1 has been demonstrated to be a marker of cellular stress and damage in infarcted regions.^70^, ^71^

#### 2.2.2 The roles that HSP20 and HSP27 play in SAH

Small heat shock proteins, including HSP20 and HSP27, are a family of low‐molecular‐weight HSPs. HSP27, also known as HSPB1, is seldom expressed in normal brain tissues and is induced mainly in astrocytes in the ischaemic zone and the ischaemic penumbra.^14^, ^40^ In contrast to HSP70, adenosine triphosphate (ATP) is not necessarily involved in the function of HSP27; moreover, HSP27 appears to solubilize misfolded proteins and hinder their aggregation rather than refold them.^40^ HSP20 was originally discovered as a byproduct of HSP27 purification, and HSP20 and HSP27 coexist in macromolecular aggregates.^72^ They are highly homologous in molecular structure, and both show high constitutive expression in smooth muscle and play a role in cerebral vasoconstriction after SAH.^73^, ^74^ Notably, phosphorylated HSP27 inhibits nucleotide‐dependent vasodilatation, while phosphorylated HSP20 promotes it.^75^, ^76^, ^77^ However, stress merely induces the expression and phosphorylation of HSP27, which accounts for impaired vasodilatation and cerebral vasospasm following SAH.^74^, ^77^ In contrast, Stetler et al demonstrated that the phosphorylation of HSP27 is essential to suppress cell death signalling and protect neurons against ischaemic injury.^78^ The ATP‐independent neuroprotective potential of HSP27 has been suggested to be critical in treating ischaemic brain injury.^14^ In addition, HSP27 and HSP20 are involved in the apoptosis pathway in numerous non‐neural systems, while their explicit roles in neural systems require further study.^14^, ^73^ A possible mechanism by which HSP20 mediates vasodilatation is shown in Figure 2.^28^

#### 2.2.3 The role of HSP90 plays in SAH

HSP90 participates in the folding, maturation and homoeostasis of cellular proteins.^79^, ^80^ Studies have shown the facilitation of HSP90 in the development of apoptosis, inflammation and BBB destruction after ischaemic stroke^81^, ^82^, ^83^; and being essential for the stabilization and function of the ligand‐gated non‐selective cation channel, the P2X7 receptor, which is abundant in nervous system and associated with the pathophysiological process of inflammation and oxidative stress underlying EBI.^84^, ^85^ A recent study showed HSP90 played a similar role in SAH and was up‐regulated 2.5 times in both the microglia and neurons in the hippocampus 48 hours post‐SAH.^79^

### 2.3 HSPs as potential therapeutic targets for SAH

Anti‐vasospasm agents have always been a critical research focus because delayed cerebral vasospasm has long been considered an essential prognostic factor, while protection against the pathophysiology processes of EBI has also become a research focus in recent years. Nimodipine, an oral calcium channel blocker first introduced in 1985, was the drug typically used to treat SAH and was suggested to effectively prevent neuroischaemic events after SAH.^22^, ^23^, ^28^, ^86^ A recent randomized trial showed that the intrathecal application of nimodipine not only reduces DCI, but also improves clinical outcome and that this approach is safe.^87^, ^88^ Other vasodilators, such as fasudil, clazosentan and nicardipine, have also been shown to have some potential value for the treatment of vasoconstriction, although they may induce systemic hypotension and cerebral perfusion pressure.^22^, ^28^

Atorvastatin was suggested to ameliorate cellular apoptosis in EBIin a rat model, which might because of the inhibition of caspase‐3 and ER stress‐related proteins such as GRP78.^46^ Although the clinical value of atorvastatin targeting EBI remains unknown, the clinical application of statin in ameliorating DCI was widely researched. A retrospective study and a prospective cohort study have shown there is a correlation between statin treatment and the decrease of vasospasm incidence after SAH.^89^, ^90^ Moreover, randomized controlled phase II studies have demonstrated that acute administration of statin directly after SAH decreased the incidence of radiological vasospasm, and reduced the clinical signs of delayed ischaemic neurological deficits and mortality.^91^, ^92^ However, some experimental and clinical studies demonstrated the different or even contradictory results.^93^, ^94^, ^95^ Thus, further well‐designed multicentre randomized controlled trials are required.

Numerous emerging agents from experimental studies also show the therapeutic potential in SAH. The oral administration of geranylgeranylacetone is reportedly capable of inducing HSP70 expression and ameliorating DCI, although its actual clinical value remains unclear.^37^ Experimental studies have also shown that, when administered intravenously, modified HSP70 proteins, for example, HSP70 attached to an antibody or terminal transactivator of transcription (TAT) motif, are capable of crossing the BBB and subsequently lead to a decrease in focal cerebral ischaemia and a better outcome.^96^, ^97^, ^98^ In addition, HO‐1 protein combined with protein transduction domains significantly attenuated cerebral vasospasm when injected into the cerebral arteries of rats.^64^ The therapeutic potential of HO‐1 inducers, including nicaraven, argon, carnosol, ebselen and CGS26393, has also been revealed.^39^, ^65^, ^71^, ^99^, ^100^, ^101^ Additionally, nicaraven is known to cause minute side effects during treatment.^65^The intravenous administration of AZX100, a molecule constructed by combining phosphopeptide mimetics of HSP20 with a cell‐permeant peptide, was proven to successfully cross the BBB and prevent and reverse the decrease in cerebral perfusion without systemic hypotension even though the dose administered was 15‐fold higher than the minimally effective dose.^28^

Valproic acid reportedly exerts neuroprotective effects in EBI.^24^ By increasing HSP70 induction, valproic acid reduces the activity and expression of MMP‐9 and prevents the degradation of claudin‐5 and occludin, thus attenuating BBB disruption and brain oedema. On the other hand, valproic acid can inhibit neuronal cell apoptosis through phosphorylated anti‐apoptotic Akt kinase, the phosphorylation of which is regulated by HSP70 (Figure 2).^24^ Furthermore, because the HSP90 or the P2X7 receptor inhibition exerts neuroprotective effects, both the 17‐allylamino‐17‐demethoxygeldanamycin and A438079, which are specific inhibitors of HSP90 and P2X7, respectively, showed the therapeutic potential in SAH mice.^79^

Unfortunately, nearly all the new drugs developed in the last few decades to alleviate vasospasm led to no better outcome, and, still worse, no clinical treatment found significant amelioration in EBI.^22^, ^39^ Additionally, Leclerc et al suggested that all the currently available models of SAH might lead to considerable variation in results and make experimental findings difficult to convert into clinical outcome owing to the lack of spontaneous aneurysm rupture.^59^ Thus, more studies targeting cerebral vasospasm or EBI and experimental models more comparable to human SAH are warranted.

## 3 THE PATHOPHYSIOLOGY OF ICH

ICH leads to destructive outcome with high mortality and morbidity.^6^, ^102^ However, there are fewer therapeutic strategies for ICH than for other types of craniocerebral vascular disorders, such as ischaemic stroke and SAH.^103^, ^104^ Some studies have demonstrated that the mortality rate of ICH has not decreased for several decades.^8^, ^105^ Therefore, investigating the pathophysiology of ICH and exploring potential targets for ICH therapy are crucial in improving the outcome of ICH.

ICH‐associated cerebral injury includes primary injury and secondary injury. Primary injury occurs immediately after bleeding and is mainly because of haematoma expansion and the physical disruption of the brain's cellular architecture caused by haematoma.^106^ Based on this mechanism, several studies have investigated the benefit of haematoma removal and surgical decompression for ICH. However, the effect of surgical treatment for ICH is still controversial. Some studies have shown that the timely removal of haematomas is important to inhibit further damage.^107^ A clinical trial showed that surgery early in the progression of ICH might confer a small but clinically relevant survival advantage for ICH, excluding intraventricular haemorrhage.^108^ However, other studies have shown no benefit of clot removal surgery in ICH.^109^

Secondary injury during ICH follows primary injury and includes the inflammatory response to haematoma, the release and accumulation of haematoma components, oxidative stress and cytotoxicity.^10^, ^105^, ^107^ Among the pathophysiology of secondary injury, inflammation is considered to be a critical part of the whole brain injury process.^105^, ^110^, ^111^, ^112^ The presence of blood components in the parenchyma of the brain induces and triggers the inflammatory response, including the release of inflammatory mediators, enzyme activation, inflammatory cell migration and the breakdown and repair of brain tissue, which aggravates ICH damage through complicated pathways and leads to irreversible effects such as brain barrier disruption, brain oedema and brain cell death.^10^, ^110^, ^113^, ^114^ As the treatment for primary injury is time‐limited and still controversial, understanding the mechanism of secondary injury, especially inflammation, in ICH and exploring potential therapeutic targets is a method anticipated to improve the outcome of ICH.

### 3.1 The role of HO‐1 in ICH

As mentioned previously, HO‐1 is an isoenzyme of haeme oxygenase that catalyzes the rate‐limiting step in the metabolism of haeme and has been proven to play a neuroprotective role in SAH. Also, HO‐1 levels have already been proven to be associated with ICH.^115^ Unfortunately, although the role of HO‐1 in ICH has been researched extensively, the effect of HO‐1 in ICH is still controversial. Most scholars believe that the overall effect of HO‐1 in ICH tends to be harmful.^14^ HO‐1 metabolizes haeme to ferrous iron, biliverdin and CO. After ICH induction, the pro‐oxidant hydroxyl radical induced by the reaction of ferrous iron and hydrogen peroxide increases oxidative stress, and HO‐1 accelerate the accumulation of iron overload in the brain, both of which are involved in the HO‐1 induced damage.^14^, ^116^, ^117^ Moreover, a study proving the harmful role of HO‐1 demonstrated a marked reduction of ICH‐induced leucocyte infiltration and microglia/macrophage activation in HO‐1 knockout mice, which implied a function of HO‐1 in promoting inflammation and increasing secondary injury.^116^

**Figure 3:**
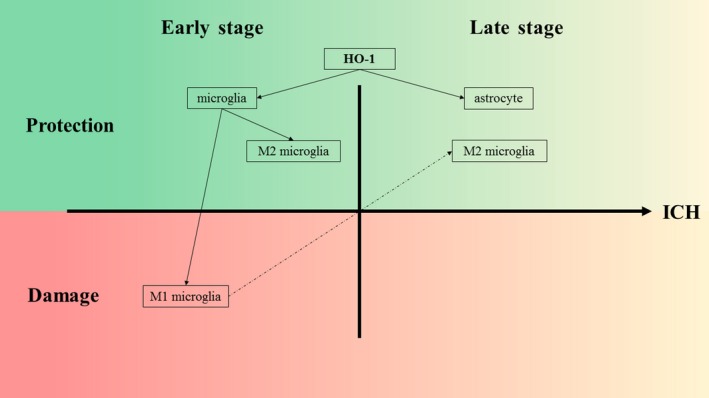
The expression of HO‐1 after ICH. In the early stage after ICH, HO‐1 is mainly expressed in microglia, and the activation of microglia leads to the development of M1‐like or M2‐like phenotypes. The former contributes to neurological impairments, while the latter is neuroprotective. Furthermore, the two subtypes of microglia are in dynamic flux after ICH. In the late stages of ICH, the overexpression of HO‐1 in astrocytes is neuroprotective and reduces blood‐brain barrier disruption, perihaematomal cell injury and mortality following ICH. M1 microglia, M1‐like phenotype of microglia; M2 microglia, M2‐like phenotype of microglia

Further studies revealed that HO‐1 levels fluctuate after ICH, and the effect of HO‐1 after ICH depends on the time‐point of the ICH process.^118^ In the early stage after ICH, HO‐1 is mainly expressed in microglia, the major phagocytes in the brain, and the activation of microglia contributes to neurological impairment.^119^ Furthermore, the activation of microglia leads to the development of M1‐like or M2‐like phenotypes in the microglia, which are in dynamic flux after ICH.^120^, ^121^ Between these two phenotypes, M1 microglia produce pro‐inflammatory factors that then contribute to brain damage, while M2 microglia induce anti‐inflammatory factors and are neuroprotective.^122^ Moreover, HO‐1 is also expressed in astrocytes in the late stages of ICH, and many studies have proven that HO‐1 overexpression in astrocytes provides neuroprotection and reduces brain barrier disruption, peri‐haematoma cell injury and mortality after ICH (Figure 3).^123^, ^124^, ^125^, ^126^ One of these studies revealed that HO‐1 induction could protect cortical astrocytes from haemoglobin toxicity,^126^ which may be one of the mechanisms of the neuroprotective function of HO‐1. The mechanisms described above may explain the different effects of HO‐1 at different time‐points of ICH. In contrast, another study showed that HO‐1 was up‐regulated in the early stage after ICH and exerted a protective effect against oxidative stress; however, in the late stage of ICH, the expression of HO‐1 may result in neurological dysfunction, and HO‐1 may be toxic.^118^

Besides microglia, there are other types of tissue‐resident macrophages that may participate in different stages of ICH. Border‐associated macrophages (BAMs) in brain contain cells locating in the perivascular spaces of brain vessels, the leptomeningeal spaces and the choroid plexus, which have scavenger function and the ability to present antigen to lymphocytes.^127^ Although a recent study has shown BAMs attract granulocytes and promote vascular leakage after ischaemic stroke, it is still little known about the function of these macrophages under the ICH conditions.^128^ But researchers believe that perivascular macrophages and meningeal macrophages have been identified by the expression of CD163 in both humans and rats.^129^, ^130^ CD163 is up‐regulated and participates in haemoglobin clearance after ICH and may be a biomarker of functional outcome in ICH.^131^, ^132^, ^133^ A previous study has proved that CD163/HO‐1 pathway could regulate inflammation in haematoma surrounding tissues after ICH; specifically, about 24 hours after ICH onset, CD163 and HO‐1 expressions would be gradually enhanced and reach a peak after 72 hours, exhibiting anti‐inflammatory characteristics.^134^

### 3.2 The role of HSP70 in ICH

As indicated in the paragraphs above, HSP70 is a common and extensively studied HSP. The relationship between HSP70 and ICH has also been clarified. Studies demonstrated that HSP70 levels changed in patients with intracranial haemorrhage and were negatively associated with Glasgow Coma Scale scores and positively associated with bleeding volume,^36^, ^135^ and HSP70 is proven to ameliorate the neurological deficits in the delayed but not acute phase of ICH.^136^ The function of glucose‐regulated protein 75 (GRP75) and GRP78 in ICH have also been examined in many studies.

#### 3.2.1 GRP75 and ICH

Glucose‐regulated protein 75 is a member of the HSP70 family and is mainly located in mitochondria.^137^ As GRP75 showed a potential protective effect in central nervous system disorders,^138^, ^139^ its role in ICH was also studied. In a rat ICH model, GRP75 expression was reduced upon ICH, and further research demonstrated that GRP75 overexpression in brain tissues with ICH inhibited inflammatory factors such as tumour necrosis factor‐α and interleukin‐1β and inhibited the expression of neuronal apoptosis markers (such as active caspase‐3 and B cell lymphoma 2 apoptosis regulator‐associated X apoptosis regulator).^140^ These results showed the protective role of GRP75 in ICH, which includes the inhibition of inflammation and neuronal apoptosis.

#### 3.2.2 GRP78 and ICH

Glucose‐regulated protein 78 was also reported to be associated with ICH. A study of the role of a 45‐kD Ca^2+^‐binding protein demonstrated that the interaction of the Ca^2+^‐binding protein and GRP78 led to the inhibition of neuronal apoptosis.^141^ In addition, ER stress pathway is also involved tightly in cell apoptosis of secondary injury after ICH.^142^, ^143^ Thus, similar to GRP75, the role of GRP78 in ICH may be protective for brain tissues. However, additional studies on GRP78 and ICH are required.

### 3.3 Therapeutic strategies targeting HSPs in ICH

According to the role of HSPs in ICH, some therapeutic strategies targeting HSPs are potential methods to improve ICH outcome. For HO‐1, which is a key enzyme of haeme metabolism, the HO‐1 inducer haemin was proven to improve BBB function and neurological outcome in ICH models.^20^ Furthermore, nuclear factor erythroid‐2 related factor 2 (Nrf2), which can regulate the expression of HO‐1 and many other proteins with antioxidant and anti‐inflammatory effects, was shown to be a target for therapy in ICH (Figure 2).^144^ Therefore, Nrf2 activators targeting the Nrf2/HO‐1 axis are neuroprotective in ICH and may be potential drugs for ICH treatment. For instance, a study focusing on nicotinamide mononucleotides demonstrated that nicotinamide mononucleotides activated the Nrf2/HO‐1 pathway to attenuate brain injury after ICH.^145^ In addition, minocycline was proven to significantly reduce iron overload and iron handling proteins induced by HO‐1 and also reduce brain swelling, inflammation and neuronal loss, leading to a better outcome for ICH in a rat model.^146^

Similarly, a high level of HSP70 was detected in minocycline‐treated rats with ICH, which showed that minocycline exerted a neuroprotective effect by mediating HSP70.^147^, ^148^ Another study demonstrated that geranylgeranylacetone, which is known to be an HSP70 inducer, could reduce brain oedema and exerts neuroprotective effects in an ICH model.^149^ Administration of another HSP inducer geldanamycin, also resulted in amelioration of inflammation, neurobehavioral deficits and BBB destruction by the up‐regulation of HSP70.^136^ Moreover, mild hypothermia exerted a protective effect on rat neuronal injury after ICH by reducing ER stress, in which GRP78 plays a vital role.^142^, ^143^ Similarly, a kind of Chinese traditional medicine, Di Dang Tang, which involves four traditional Chinese medicinal substances, was proven to block the GRP78‐IRE1/PERK pathway post‐ICH and results in reduced ER stress‐mediated apoptosis (Figure 2).^150^

## 4 CONCLUSIONS

In the present study, we focus on the current understanding of the role and therapeutic potential of HSPs involved in haemorrhagic stroke. HSP70 acts as a critical neuroprotective molecule and participates in the processes of cerebral vasospasm, neural cell apoptosis, immunoreaction and inflammation during haemorrhagic stroke. Additionally, HSP70 is a significant marker for cellular stress or damage and can be used for outcome prediction and differential diagnosis. HO‐1 is capable of enhancing haeme clearance, intensifying iron sequestration and increasing the antioxidant bilirubin after haemorrhage; however, the metabolic process can be toxic because of iron overload and the succedent pathway, for example, the Fenton reaction and oxidative damage, and the overall effect of HO‐1 in SAH and ICH tends to be protective or harmful, respectively, given their different pathophysiological mechanisms. In addition, the role of HO‐1 in ICH was proven to vary at different stages during the ICH process, and further research is needed to explore the effects of HO‐1 in ICH. HSP20 and HSP27 mainly play a role in SAH. Despite being highly homologous in molecular structure, the phosphorylation of HSP20 and HSP27 mediates opposite impacts on SAH, as the former promotes nucleotide‐dependent vasodilatation, and the latter inhibits it. Even so, the ATP‐independent neuroprotective potential of HSP27 is suggested to be critical in ischaemic brain injury. Pathophysiological processes following initial haemorrhage present a high risk of disability and mortality among survivors, and new drugs developed over the years have failed to improve the clinical outcome of patients. HSPs represent potential therapeutic targets, and new agents targeting HSPs are warranted.

## CONFLICTS OF INTEREST

The authors declare that they have no competing interests.

## AUTHOR CONTRIBUTION

AWS, YXZ and YHY wrote the paper and made the original figures. WHZ, JMZ and YCD critically revised the texts and figures. All authors read and approved the final manuscript.
